# RSPO3 is a prognostic biomarker and mediator of invasiveness in prostate cancer

**DOI:** 10.1186/s12967-019-1878-3

**Published:** 2019-04-15

**Authors:** Aruz Mesci, Fabrice Lucien, Xiaoyong Huang, Eric H. Wang, David Shin, Michelle Meringer, Christianne Hoey, Jessica Ray, Paul C. Boutros, Hon S. Leong, Stanley K. Liu

**Affiliations:** 1 Sunnybrook Research Institute, Sunnybrook Health Sciences Centre, Toronto, ON Canada; 2 Department of Radiation Oncology, University of Toronto, Toronto, ON Canada; 3 Mayo Clinic Cancer Centre, Rochester, MN USA; 4 Department of Medical Biophysics, University of Toronto, Toronto, Canada; 5 Ontario Institute for Cancer Research, Toronto, ON Canada

**Keywords:** RSPO3, Prostate cancer, Invasion, Biochemical relapse

## Abstract

**Background:**

While prostate cancer can often manifest as an indolent disease, the development of locally-advanced or metastatic disease can cause significant morbidity or mortality. Elucidation of molecular mechanisms contributing to disease progression is crucial for more accurate prognostication and effective treatments. R-Spondin 3 (RSPO3) is a protein previously implicated in the progression of colorectal and lung cancers. However, a role for RSPO3 in prostate cancer prognosis and behaviour has not been explored.

**Methods:**

We compare the relative levels of RSPO3 expression between normal prostate tissue and prostate cancer in two independent patient cohorts (Taylor and GSE70768—Cambridge). We also examine the association of biochemical relapse with RSPO3 levels in these cohorts. For elucidation of the biological effect of RSPO3, we use siRNA technology to reduce the levels of RSPO3 in established prostate cancer cell lines, and perform in vitro proliferation, invasion, western blotting for EMT markers and clonogenic survival assays for radiation resistance. Furthermore, we show consequences of RSPO3 knockdown in an established chick chorioallantoic membrane (CAM) assay model of metastasis.

**Results:**

RSPO3 levels are lower in prostate cancer than normal prostate, with a tendency for further loss in metastatic disease. Patients with lower RSPO3 expression have lower rates of biochemical relapse-free survival. SiRNA-mediated loss of RSPO3 results in no change to clonogenic survival and a lower proliferative rate, but increased invasiveness in vitro with induction of epithelial–mesenchymal transition (EMT) markers. Consistent with these results, lower RSPO3 expression translates to greater metastatic capacity in the CAM assay. Together, our preclinical findings identify a role of RSPO3 downregulation in prostate cancer invasiveness, and provide a potential explanation for how RSPO3 functions as a positive prognostic marker in prostate cancer.

**Electronic supplementary material:**

The online version of this article (10.1186/s12967-019-1878-3) contains supplementary material, which is available to authorized users.

## Introduction

Prostate cancer is the second most common malignancy of males, and the fifth leading cause of cancer death worldwide [1]. An estimated 1.1 million men were diagnosed with prostate cancer in 2012 across the world [1]. While this disease can follow an indolent course, a substantial number of patients develop locally advanced or metastatic disease with associated morbidity and mortality. Currently, management paradigms are largely guided by risk strata based on clinical stage, Gleason score, and serum prostate-specific antigen (PSA) test. A better understanding of the molecular pathways contributing to disease progression is necessary to improving our current diagnostic, prognostic and therapeutic capabilities. Wnt constitutes one such pathway that is of great interest to the prostate cancer community.

Wnt family comprises several secreted glycoproteins that can bind to several Frizzled (FZD) transmembrane receptors, resulting in activation of one of the two principal Wnt pathways (i.e. “canonical” or “non-canonical”) [2]. In the canonical pathway, Wnt activation results in rescue of β-catenin molecule from degradation by a molecular complex containing glycogen synthase kinase-3 (GSK3), casein kinase-1 (CK1), axin, and adenomatous polyposis coli (APC). As a result, β-catenin translocates to the nucleus, and binds to T cell factor–lymphoid enhancer factor (TCF–LEF) transcription factors, as well as co-activators, resulting in transcription of Wnt target genes. On the other hand, non-canonical pathway includes multiple parallel pathways that act through several mechanisms including small GTPases and calcium channels/protein kinase C (PKC). Functionally, contribution of the Wnt pathway to prostate cancer is incompletely understood. Upregulation of various players in Wnt signaling (including Wnt ligands, receptors, and regulator molecules) has been observed in certain prostate tumours or stroma [2]. However, there exist conflicting reports regarding possible mechanisms and biological significance of these changes. For instance, interaction between androgen receptor (AR) and Wnt signaling has been proposed by several groups [3], but certain groups propose an inhibitory interaction between the two pathways [4], while others propose a synergistic relationship [5]. Similarly, it has been proposed that WNT5a may act as a tumour suppressor role in that its expression may induce apoptosis in prostate cancer cell lines [6] and predict for increased biochemical recurrence-free survival [7, 8]. However, other data suggests that WNT5a expression may increase markers of epithelial-to-mesenchymal transition, and correlate with higher Gleason grade disease [9]. In addition to the complexity contributed by conflicting roles of the same WNT ligand, there also exist other Wnt ligands with potentially separate biological contributions to prostate cancer. For example, WNT11 expression appears to be upregulated by androgen depravation treatment (ADT) [10], and WNT11 expression may promote neuroendocrine differentiation in prostate cancer [11]. Given the multitude of molecules and several distinct pathways, the place of Wnt signaling in prostate cancer remains unclear. Studies on specific Wnt ligands or their regulators may, thus, represent a more feasible option to appreciate the role of this pathway.

R-Spondin 3 (RSPO3/PWTSR/THSD2/CRISTIN1) is a secreted protein belonging to the R-Spondin family that acts as an activator of Wnt/β-catenin pathways [12]. RSPO3 was first identified as a Thrombospondin superfamily protein cloned from fetal brain RNA, with a thrombospondin superfamily repeat (TSR) domain [13]. The authors noted that RSPO3 transcripts were ubiquitously expressed in normal tissue, including lung and ovary, but missing in GI-107 ovarian and LX1 lung tumour cell lines. Wnt activation by RSPO3 is thought to occur via both canonical and non-canonical pathways [14]. The canonical pathway has been suggested to occur via Leucine-rich repeat-containing G-protein coupled receptors 5 and 6 (LGR5 and LGR6) in a clathrin endocytosis-dependent fashion [14, 15]. Conversely, the non-canonical pathway may occur through Wnt/PCP pathway in cooperation with WNT5A and Syndecan-4 [14, 16]. Several reports outline various roles for RSPO3 in normal metabolism and development. For instance, RSPO3 is implicated in angiogenesis, with *Rspo3*^−*/*−^ knockout mice displaying embryonic lethality at day 10 due to failure of fetal blood penetration into the chorion [14, 17]. Beyond early embryonic stage, deletion of RSPO3 has been shown to result in lethality due to a lack of appropriate cardiac development [18]. RSPO3 is also thought to regulate myogenesis and limb formation; significant defects in myogenic differentiation/myotubule [19] and hindlimb formation [20] are observed when RSPO3 is co-deleted with RSPO2, another R-Spondin family member. In humans, RSPO3 is implicated in genome-wide association studies examining single nucleotide polymorphisms (SNPs) in other metabolic functions such as fat distribution [21], and bone mineral density/fracture risk [22], Specific role of RSPO3 in these contexts is not yet fully elucidated. Several reports address the contribution of RSPO3 in oncology also.

One of the first reports to link RSPO3 with cancer showed RSPO3 to be frequently upregulated by genomic integration of the mouse mammary tumour virus (MMTV) [23]. In the same report, normal mouse mammary tumours transduced to express RSPO3, but not the empty vector controls, produced tumours. In a subsequent study, Runx1 was shown to bind to the RSPO3 promoter region and upregulate its expression in mouse mammary tumours [24]. Nonetheless, the generalizability of these findings in human breast cancer remains uncertain, as gene expression analysis in a cohort of breast cancer patients from France did not show RSPO3 overexpression to occur with an appreciable frequency [25] (1/446 patients). The oncogenic role of RSPO3 has been most extensively studied in colorectal carcinoma (CRC). In an early report, recurring RNA transcript fusions of RSPO3 with protein tyrosine phosphatase, receptor type kappa (PTPRK) were noted in a subset of primary human CRC samples [26]. When these RSPO3–PTPRK fusions were targeted using antibodies generated against RSPO3 in xenografts, tumour growth was inhibited [27]. Consistent with a pro-oncogenic role, generation of RSPO3–PTPRK fusions in mouse intestinal epithelia were sufficient to initiate intestinal hyperplasia and tumorigenesis [28, 29]. This process has been shown to be Wnt-dependent, as blockade of Wnt secretion by inhibition of Porcupine resulted in a loss of cell proliferation, reduction of tumour volume, as well as a change of global gene expression profile consistent with a more differentiated state. On the other hand, RSPO3–PTPRK fusions may not necessarily play a role in other cancers remains, since reports in ovarian cancer and lung adenocarcinoma have failed to show this aberrant gene fusion [30, 31]. Little has been reported regarding patient outcomes according to RSPO3 expression status; however, one report described RSPO3 overexpression in a subset of primary lung adenocarcinoma samples, with poorer overall survival in patients with higher expression [31]. Interestingly, this association may be histopathology-specific, as a separate study showed RSPO3 downregulation in squamous cell lung cancer, but upregulation in lung adenocarcinoma, compared to normal lung tissue [32]. Taken together, these studies suggest a potentially tumour-promoting role for RSPO3 in a tissue-specific fashion. Despite these reports on a variety of cancers, studies on a potential role of RSPO3 in prostate cancer are conspicuously missing. In this report, we show that RSPO3 expression is lower in prostate cancer versus normal tissue, and that lower levels of RSPO3 prognosticate reduced biochemical relapse-free survival in several patient cohorts. Using in vitro experiments as well as a validated ex ovo assay, we show that RSPO3 loss translates into greater invasiveness and extravasation by tumour cells.

## Materials and methods

### Bioinformatic analyses

Oncomine Platform (http://www.oncomine.org) was used with the following parameters to identify available datasets containing RSPO3 gene expression as well as biochemical relapse data. The following parameters were used: (1) Primary Filters: Gene Name—RSPO3; Differential Analysis—cancer vs. normal analysis; Cancer type—prostate cancer. (2) Sample Filters: Clinical Outcome—Recurrence Status. PROGgene V2 Prognostic Database (http://watson.compbio.iupui.edu/chirayu/proggene/database/?url=proggene) was used in parallel to identify other datasets that include RSPO3 expression and relapse-free survival information. The following parameters were used: Single user input genes—RSPO3; Cancer Type—Prostate Cancer; Survival Measure—Relapse. RSPO3 gene expression profile and biochemical relapse-free survival information was then independently extracted from the resulting publicly available datasets. A schematic diagram outlining our search strategy is provided (Additional file 1: Figure S1). Statistical analyses were performed as described below.

### Cell lines and cell culture

Human prostate adenocarcinoma (DU145 and 22RV1) cell lines were purchased from American Type Culture Collection (ATCC; VA, USA). Cells were maintained in their respective culture media (Dulbecco’s Modified Eagle’s Medium—DMEM for DU145 and Roswell Park Memorial Medium 1640—RPMI for 22RV1) with supplements as below. Early passage cell lines were cultured in media supplemented with 10% fetal bovine serum (FBS) (Invitrogen, Ontario, Canada) and penicillin (100 U/mL)—streptomycin (100 μg/mL) (Invitrogen, Ontario, Canada), and maintained in a humidified 37 °C incubator with 5% CO_2_. 22RV1 cells were maintained in Roswell Park Memorial Medium 1640 (RPMI 1640) supplemented with 10% fetal bovine serum (FBS) (Invitrogen, Ontario, Canada) and penicillin (100 U/mL)—streptomycin (100 μg/mL) (Invitrogen, Ontario, Canada). Media are hereafter referred to as 10% DMEM or as 10% RPMI. Cell lines were passaged when they reached approximately 80% confluency and were regularly tested with MycoAlert (Lonza, Ontario, Canada) to ensure the absence of mycoplasma contamination.

### Transfection of siRNA and cDNA

3 × 10^5^ cells were seeded into 6-well plates, then 16 h later, control or a pool of 3 different siRNA for RSPO3 (Santa Cruz Biotechnology, CA, USA) were transiently transfected into cells using Lipofectamine 2000 (Invitrogen, Ontario, Canada) as per manufacturer’s recommendations. Similarly, overexpression experiments were carried out with the same protocol above, using empty pCMV6-XL5 vector, or the same vector containing the human RSPO3 ORF cassette (Origene, Rockville, MD). In vitro assays (described below) were performed 24 h later on the transfected cells.

### Generation of stable cell lines overexpressing short hairpin RNA specific for RSPO3

Cells were transduced with neomycin-selectable vectors expressing shRSPO3 or non-silencing control as per manufacturer’s instructions (ThermoScientific, PA, USA), selected using a neomycin for 2 weeks and separate colonies of stable transductants were grown and tested for RSPO3 expression using RT-PCR.

### Real-time quantitative PCR

For gene expression analysis, RNA was extracted using the RNeasy Mini kit (Qiagen, Ontario, Canada) and cDNA synthesized using Omniscript RT kit (Qiagen, Ontario, Canada) as per manufacturer’s instructions. RSPO3 expression levels were quantified through quantitative real-time PCR using the QuantiTect SYBR Green PCR kit (Qiagen, Ontario, Canada) on the StepOnePlus Real-time PCR system. A three-step PCR reaction was used (94 °C × 15 s, 55 °C × 30 s, 70 °C × 30 s; 45 cycles), and mRNA levels were calculated using the comparative Ct method via StepOne Software (Life Technologies, Ontario, Canada), and relative expression levels normalized to GAPDH (for mRNA)). Primer sequences were as follows: GAPDH-F: CAGCCTCAAGATCATCAGCA; GAPDH-R:GTCTTCTGGGTGGCAGTGAT; RSPO3-F: GCCTTGACAATTGCCCAGAA:; RSPO3-R: TCCATGCACGAAGAAGGGAA. RSPO1-F: GCTCTGCTCTGAAGTCAACG; RSPO1-R: CTCACAGTGCTCGATCTTGC; RSPO2-F: GCGAATGGGGAACTTGTAGC; RSPO2-R: TCTTCTCCTTCGCCTTTGGT; RSPO4-F: CCCTGCACACACAATGGAAA; RSPO4-R: GTCCTTCCTGCCCTTCTTCT. All primers were purchased from Life Technologies (Burlington, Ontario, Canada).

### ELISA assay

A human RSPO3 sandwich ELISA kit was purchased from LifeSpan Biosciences (Seattle, WA). 5x10^5^ cells were seeded and transfected as described. Lysates were collected 48 h after transfection. Protein concentration of each lysate sample was determined by a standard curve using the Bradford assay. 600 μg of lysate (or buffer-only negative control) was loaded into each well. The ELISA procedure was carried out as instructed by supplier. A standard curve was generated using the lyophilized protein supplied. Negative control is sample diluent only. Optical density was determined using a plate reader (450 nm) as instructed by the supplier.

### Cellular proliferation assay

Cells were seeded in triplicate (0.5 × 10^5^ cells/well In 10% DMEM in 6-well plates). Four days later, cells were detached using trypsin and total viable cell number determined using the Countess automated cell counter (Life Technologies). Trypan blue dye was used in the assay for live-dead cell discrimination.

### Radiation clonogenic survival assay

Cells were seeded at 250, 500, 2000, and 4000 cells per well onto a six-well plate in 10% DMEM in triplicate and mock irradiated (0 Gy) or irradiated with 4, 6, or 8 Gy dose of ionizing radiation, respectively. Then cells were placed in a humidified CO2 incubator at 37 °C to allow colonies to form. Colonies were stained with crystal violet staining solution (0.5% crystal violet (Sigma-Aldrich), 25% methanol) and counted. Survival was expressed as the relative plating efficiencies of the treated cells compared with that of the mock-irradiated cells. The experiments were performed three separate times. Radiation dose–response curves were created by fitting the data to the linear quadratic equation S = e (− αD − βD^2^) using GraphPad Prism 5.0 (GraphPad Software Inc.), where S is the surviving fraction, α and β are inactivation constants, and D is the dose in Gy. The area under the curves (AUC) that represent the mean inactivation dose (MID) were also calculated using GraphPad Prism.

### Apoptosis assay

1 × 10^5^ cells were seeded in 6-well plates, and cells were transfected after 24 h as described. 48 h after transfection, cells were detached from the plates using trypsin, washed in phosphate-buffered saline, and then pelleted by centrifugation. Cells were resuspended in 500 μL of AnnexinV Binding Buffer (Biovision Inc, San Francisco, CA). AnnexinV antibody conjugated to Cy5 fluorophore was added at a 1:100 dilution, and cells were incubated at room temperature in the dark for 5 min. After washing, cells were collected by centrifugation, resuspended in PBS, and propidium iodide (1 mg/mL; Sigma-Aldrich) was added at a 1:1000 dilution. Thereafter, 20,000 events captured on a FACSCalibur flow cytometer (BD Biosciences) and data were analyzed using FlowJo 10.0.4 (Tree Star Inc.).

### Cell cycle analysis

Cells were trypsinized, washed in PBS, and fixed in ice-cold 80% ethanol in Hank’s Buffered Salt Solution (HBSS; 137 mmol/L NaCl, 5.4 mmol/L KCl, 0.25 mmol/L Na_2_HPO_4_, 0.44 mmol/L KH_2_PO_4_, 1.3 mmol/L CaCl_2_, 1.0 mmol/L MgSO_4_, 4.2 mmol/L NaHCO_3_) for 30 min on ice. Fixed cells were collected by centrifugation, washed twice with PBS, and resuspended in 50 μg/mL propidium iodide (Sigma-Aldrich) with 0.6% NP-40 (Thermo Fisher Scientific) and 0.1 mg/mL RNAse A in HBSS for 30 min at room temperature in the dark. Cells were then collected by centrifugation, resuspended in PBS, and 20,000 events captured on a FACSCalibur flow cytometer (BD Biosciences) and cell-cycle profile analyzed using FlowJo 10.0.4 (Tree Star Inc.).

### Matrigel transwell invasion assay

Cells were serum starved overnight (0.1% DMEM), then 2 × 10^5^ cells were seeded on top of 8 μm transwell inserts (BD Biosciences, Ontario, Canada) with 0.1% DMEM or RPMI and pre-coated with 1 mg/mL Matrigel (Becton, Dickinson and Company, Ontario, Canada); 10% DMEM or RPMI was used as a chemoattractant. After 24 h, cells that had invaded through the Matrigel coated transwell inserts were fixed, stained by Kwik-Diff Stain (Thermo Fisher Scientific, Ontario, Canada) and number of invading cells counted under 10× using a Leica DM LB2 microscope (Leica Microsystems, Ontario, Canada).

### Western blotting

Transfected cells were lysed with NP‐40 lysis buffer, as western blot performed as previously described [33] with 40 μg lysate run per lane. All antibodies were purchased from Cell Signaling Technology, with working concentrations used as per manufacturer’s instructions. The western blotting experiment was performed in three biological replicates.

### CAM assay

The chicken chorioallantoic membrane (CAM) assay for ex ovo metastasis and extravasation efficiency was performed as previously described [34]. 22RV1 cells stably expressing shRSPO3 or the control shRNA2 were grown to ~ 80–90% confluency. Cells were labeled with Celltracker Green (Thermo Fisher Scientific, Ontario, Canada) and then resuspended in PBS at a concentration of 1.0 × 10^6^ cells/mL. 200 microliters of each group’s cell suspension were injected intravenously into the CAM of day 13 embryos (n = 12–14 per group) using a microinjector. Intravascular and extravasated cells were counted in a two different areas marked by an aluminum foil window (1 in. × 1 in.) at T = 0 h and T = 24 h respectively using wide-field fluorescence microscopy and 10× objective. At least 100 cells per region of interest were examined at T = 0 h. Extravasation efficiency of each group per embryo was calculated by dividing the number of extravasated cells at T = 24 h by the number of intravascular cells at T = 0 h. Then, mean extravasation efficiencies in the embryos were calculated.

### Statistical analysis

All statistical tests were two-sided, and the statistical analysis was performed using the GraphPad Prism version 5.0 program (GraphPad Software, CA, USA). Statistical significance was defined as p < 0.05. The Student t-test was used to compare the mean values between two groups. Data are presented as mean values with standard deviations unless otherwise noted. Kaplan–Meier curves were plotted using GraphPad Prism version 5.0 program, as well, and statistical significance was tested using the Gehan–Breslow–Wilcoxon method.

## Results

### RSPO3 expression is decreased in prostate cancer and prognosticates poorer biochemical relapse-free survival

**Fig. 1:**
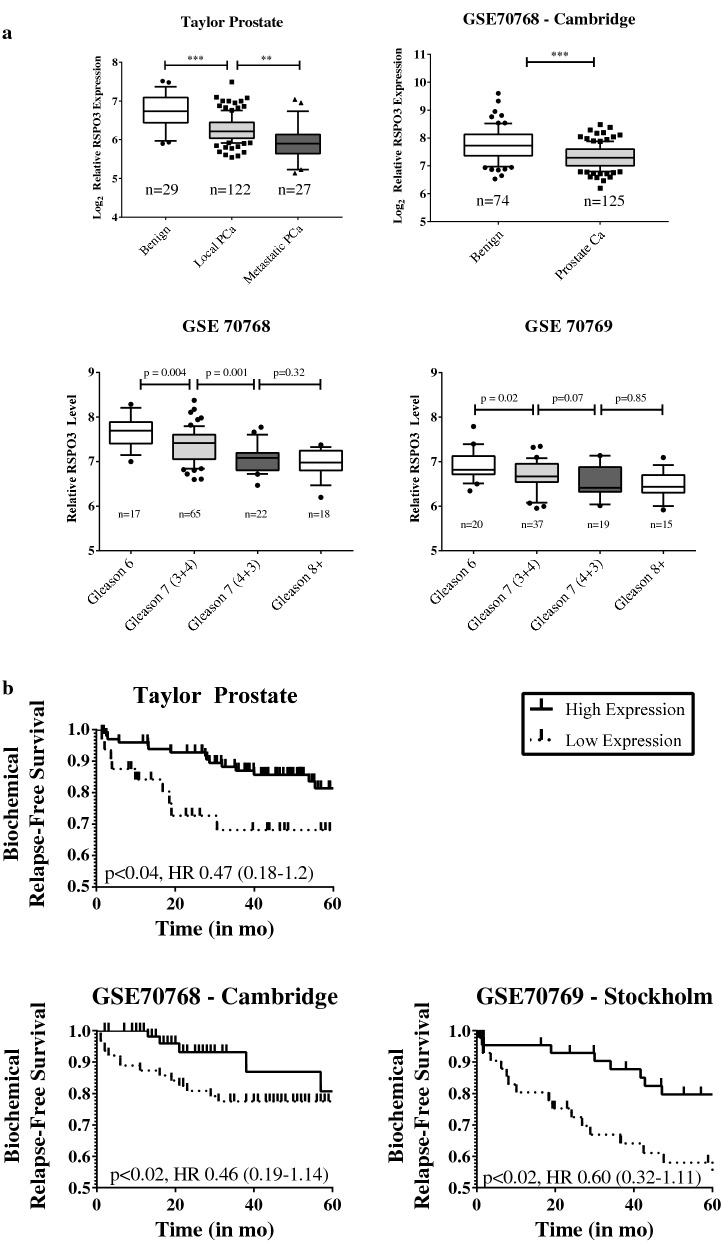
RSPO3 levels are lower in human prostate carcinoma, and RSPO3 is associated with reduced biochemical relapse-free survival. **a** Expression levels of RSPO3 in two cohorts of prostate cancer patients. Taylor Prostate (top left)—comparison of normal tissue, localized prostate cancer, and metastatic prostate cancer, and GSE70768 Cambridge (top right)—comparison of normal tissue versus prostate carcinoma) are shown. Expression levels of RSPO3 in localized prostate cancer according to Gleason grade are also given for Taylor Prostate (bottom left), and for GSE70768 Cambridge (bottom right). 25–75 percentile range is shown by the box, median is indicated by the line inside the 25–75 percentile. Also shown are 10–90 percentile range (lines above and below the 25–75 percentile), and values outside of 10–90 percentile ranges (dots). Sample sizes are indicated below. ***p < 0.001; **p < 0.01. **b** Kaplan–Meier curve of biochemical relapse-free survival in the Taylor Prostate (top left), GSE70768 Cambridge (bottom left), and GSE70769 Stockholm (bottom right; validation cohort for GSE70768 Cambridge 35) cohorts. Cohorts are divided according to RSPO3 expression. Solid line, high RSPO3 expression (higher 50 percentile—Cambridge and Stockholm; higher 75 percentile Taylor); dotted line, low RSPO3 expression (lower 50 percentile—Cambridge and Stockholm; lower 25 percentile—Taylor). A log-rank test was performed for statistical significance, and hazard ratio was calculated between the two groups (shown)

Given the previous reports outlining overexpression of RSPO3 in various cancers, we sought to investigate whether RSPO3 overexpression is observed in patient cohorts of human prostate cancer using Oncomine Platform (http://www.oncomine.com, April 2018), a repository of gene expression datasets. Furthermore, given the previous findings in lung adenocarcinoma, we limited our search to datasets containing survival information for a possible correlation of RSPO3 expression with prostate cancer survival outcomes. We identified one mRNA gene expression dataset, the Memorial Sloan Kettering Cancer Center (MSKCC)—Taylor Prostate dataset. Briefly, this dataset contains transcriptome analysis of 218 human prostate cancer tumours (181 primary and 37 metastatic) [35]. We further used ProgGene V2 Prognostic Database (http://watson.compbio.iupui.edu/chirayu/proggene/database/?url=proggene, [36]) to search for datasets not present in Oncomine. This search yielded two cohorts, the Cambridge (GSE70768) and the Stockholm (GSE70769) datasets, published as a discovery and validation datasets for transcriptome analysis of 259 men with primary prostate cancer [37]. Using these data, we compared RSPO3 expression between benign prostate tissue and prostate cancer. Contrary to the observations in other cancers, RSPO3 expression was significantly lower in prostate cancer versus normal prostate tissue (13% reduction, Taylor Prostate, Fig. 1a top panel left; 11% reduction, Cambridge; Fig. 1a top panel right). Moreover, RSPO3 levels were even lower in metastatic lesions compared to localized prostate cancer (9% reduction, Taylor Prostate, Fig. 1a left). We further substratified the Cambridge and Stockholm data according to Gleason grade and compared RSPO3 expression levels. In both cohorts, there is a general trend towards lower RSPO3 expression with greater Gleason grade (Fig. 1a bottom left and bottom right panels), despite small number of samples in each stratum. To correlate RSPO3 levels and cancer outcomes directly, we stratified men according to RSPO3 expression and compared their biochemical recurrence-free survival rates between the two strata. In all three cohorts, men with lower RSPO3 levels experienced significantly lower biochemical relapse-free survival rates (HR 0.47, Taylor Prostate, Fig. 1b top; HR 0.46, Cambridge, Fig. 1b bottom left; HR 0.60, Stockholm, Fig. 1b bottom right). Collectively, these results suggest that RSPO3 loss is correlated with increased risk of disease relapse.

### RSPO3 loss results in greater invasiveness in vitro

**Fig. 2:**
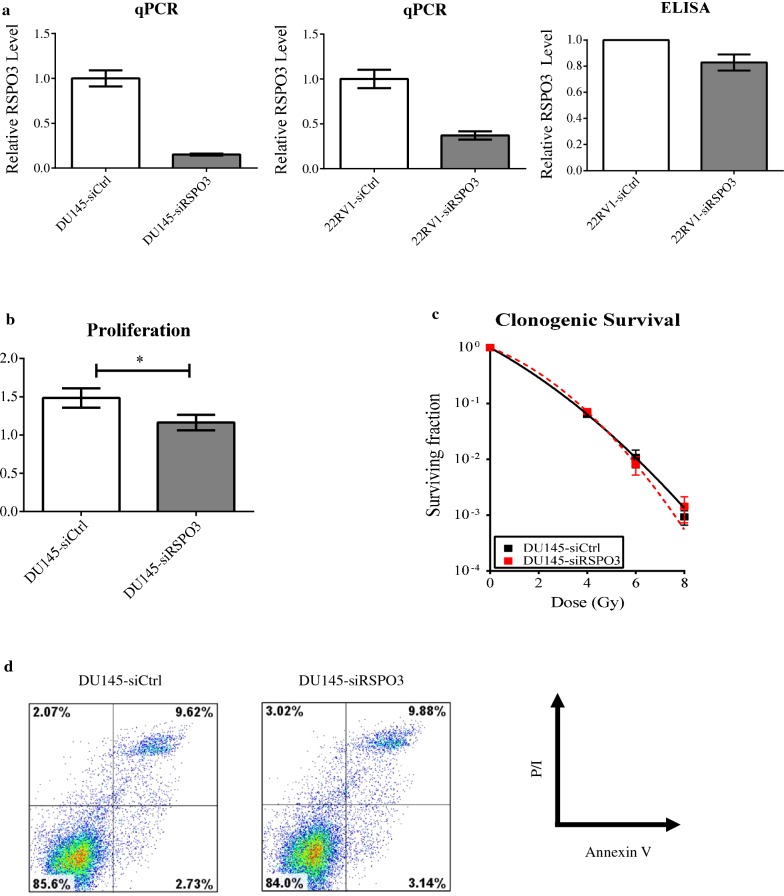
RSPO3 loss modestly decreases proliferation but significantly increases invasion. DU145 or 22RV1 cells were transiently transfected with control (siCtrl) or RSPO3-specific siRNA (siRSPO3). All figures shown are representative of 3 independent experiments. **a** qPCR and ELISA were performed for RSPO3 expression (qPCR: DU145—left, 22RV1—middle; ELISA: 22RV1—right). **b** 4 day proliferation assays were performed in transfected DU145 cells. Means and standard errors are shown. (*p < 0.05). **c** Clonogenic survival curves for radiation resistance. Surviving fraction is shown, and curves were generated using the formula: S = e (− αD − βD^2^). S, surviving fraction; α and β, inactivation constants; D, dose in Gy. (n.s.—not significant). **d** Apoptosis assay. Cells were incubated with propidium iodide and an anti-Annexin V antibody conjugated to Cy5 fluorophore, followed by flow cytometric analysis. Cells were first gated according to forward and side-scatter parameters, and fluorescence of the gated cells is shown in a 2-dimensional pseudocolour plot. Compensation controls were generated using FlowJo software using single fluorophore-stained samples. Proportion of cells in all four quadrants of the plot is given

**Fig. 3:**
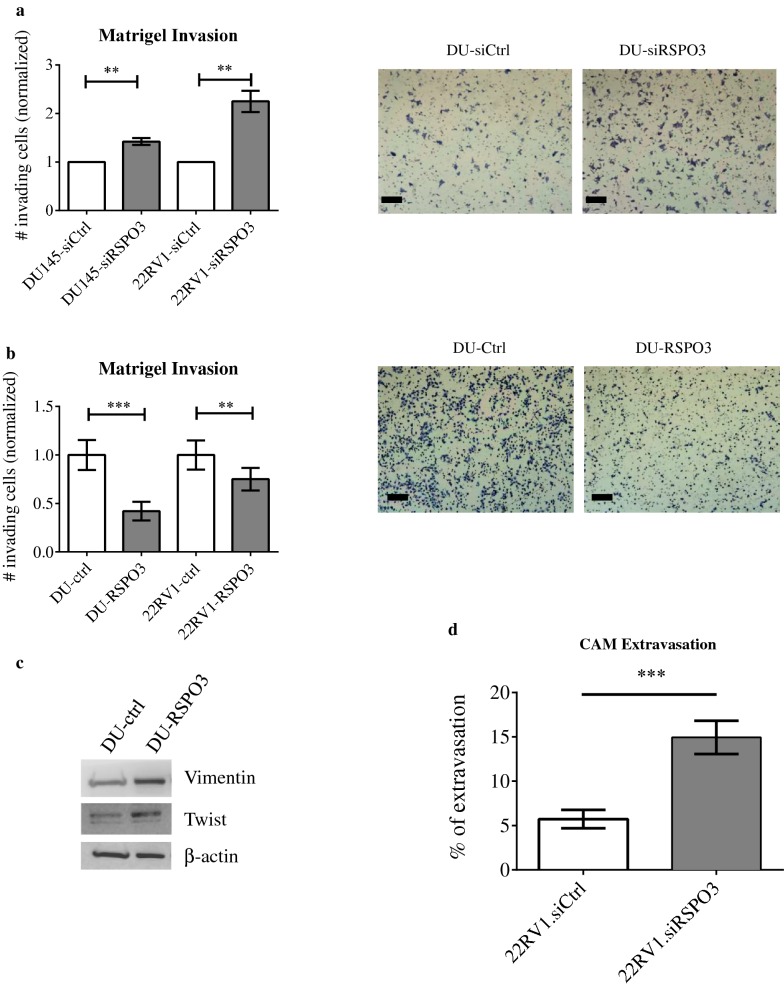
RSPO3 loss modestly decreases proliferation but significantly increases invasion. DU145 or 22RV1 cells were transiently transfected with control (siCtrl) or RSPO3-specific siRNA (siRSPO3). All figures shown are representative of 3 independent experiments. **a** Matrigel transwell invasion assay with RSPO3 knockdown. Graphs are shown with means, standard deviations, and statistical significance (left panel), and representative pictures are given with a scale bar = 20 μm, magnification: ×60. (**p < 0.01; n = 3 independent experiments). **b** Matrigel transwell invasion assays with RSPO3 overexpression. DU145 or 22RV1 cells were transfected with an empty vector (DU145-ctrl and 22RV1-ctrl) or a vector encoding a gene cassette for overexpression of RSPO3 (DU145-RSPO3 and 22RV1-RSPO3). Graphs are shown with means, standard deviations, and statistical significance (left), as well as representative images (right). Scale bar = 20 μm, magnification: ×60. (**p < 0.01; ***p < 0.001; n = 3 independent experiments). **c** Western blot for EMT markers. DU145-ctrl and DU145-RSPO3 cells were lysed and western blotting performed for the EMT markers indicated. **d** The chick chorioallantoic membrane assay for ex ovo extravasation/metastasis. 22RV1 cells were transduced with lentiviral particles carrying a vector encoding for control (22RV1.shCtrl) or RSPO3-specific (22RV1.shRSPO3) shRNA. Following selection of a colony of stable transductants, CAM assay was performed as described. Extravasation efficiency is shown with means, standard deviation, and individual measurements (dots/triangles). (**p < 0.001; n = 3 independent experiments.)

In order to characterize the functional consequences of RSPO3 loss, we obtained a panel of prostate cancer cell lines (DU145, 22RV1, LnCaP, C4-2, PC3). RT-qPCR analysis showed undetectable RSPO3 transcript levels in LnCaP, C4-2, and PC3 cell lines (ΔCT above 32; data not shown). Furthermore, given that multiple R-spondin molecules with homology exist, we tested the expression of RSPO1, 2 and 4 in DU145 and 22RV1 cells; however, these transcripts were undetectable (ΔCT above 32; data not shown). Thus, we picked DU145 and 22RV1 cells for further analysis. Firstly, we transfected these cell lines with a pool of siRNA (siRSPO3), or their scrambled controls (siCtrl) and confirmed knockdown of RSPO3 (Fig. 2a left and middle panels). We further used an ELISA assay to detect levels of RSPO3 protein in the transient transfectants, and confirmed a decrease of RSPO3 protein, though the reduction was modest (15% reduction; Fig. 2a right panel). Using the transfected cell lines, we performed in vitro assays for invasiveness (Matrigel), proliferation, anchorage-independent survival (soft agar), radiation resistance (clonogenic survival). Paradoxically, RSPO3 knockdown resulted in a small but statistically significant reduction in their proliferative capacity using a trypan blue dye-based assay (Fig. 2b; 26 ± 3% decrease). On the other hand, RSPO3 loss conferred no significant change in soft agar (not shown), clonogenic survival (Fig. 2c), or apoptosis (Fig. 2d) assays. Flow cytometric analysis of transfectants showed a decrease in S and G2/M populations (Additional file 1: Figure S2). In contrast, decrease in RSPO3 levels resulted in a significant increase in Matrigel invasion (Fig. 3a; DU145: 1.45 ± 0.04 fold increase; 22RV1: 2.15 ± 0.1-fold increase). To exclude potential pleiotropic effects of siRNA-mediated knockdown, we also utilized a mammalian overexpression vector (pCMV6XL5) containing the human RSPO3 ORF, or an empty vector. We confirmed overexpression using an ELISA assay (data not shown). In concordance with our earlier findings, RSPO3 overexpression caused a decrease in invasiveness (Fig. 3b; DU145: a decrease of 58 ± 3%; 22RV1: a decrease of 25 ± 3%). Epithelial–mesenchymal transition (EMT) is implicated in cancer cell invasion, progression and metastases, and thus we performed western blotting to assay induction of EMT markers in DU145 cells. We discovered that knockdown of RSPO3 in DU145 cells resulted in increased protein expression of vimentin and twist1, which are cytoskeletal and transcription factors, respectively and characteristic of EMT (Fig. 3c). Other EMT markers were unchanged (data not shown). Collectively, our results support a role for RSPO3 in mediating EMT and invasiveness in prostate cancer.

### Lower levels of RSPO3 confer greater invasiveness ex ovo

Given our observations in vitro, we employed a validated ex ovo model, the chick chorioallantoic membrane assay, on metastasis formation and extravasation to test the effect of RSPO3 loss [34]. To avoid possible consequences of fluctuations in RSPO3 transcript levels with transient siRNA transfection, we generated 22RV1 expressing the shRNA specific for RSPO3 (22RV1.shRSPO3) or their scrambled controls (22RV1.shCtrl) and confirmed knockdown (data not shown). Similar to our in vitro experiments, 22RV1.shRSPO3 cells displayed higher rates of extravasation in CAM assay compared to 22RV1.shCtrl cells (Fig. 3d; 5.7 ± 1.0% versus 15.0 ± 1.9% extravasation; a 2.6-fold increase), further supporting a tumour-suppressor role for RSPO3 in prostate cancer.

## Discussion

In this report, we provide evidence supporting a tumor-suppressive role for RSPO3. RSPO3 levels are lower in prostate cancer samples compared to healthy prostate samples in several patient cohorts, and lower levels of RSPO3 prognosticate poorer biochemical relapse-free survival in these cohorts. These observations in clinical samples are supported by in vitro invasion and in vivo metastasis (CAM) assays. Taken together, our results strongly suggest a tumour-suppressor role for RSPO3 in prostate cancer.

To our knowledge, our report is the first to describe a role for RSPO3 in prostate cancer. Most reports on the role of RSPO3 in cancer are derived from observations in colorectal cancer, where aberrant RSPO3–PTPRK fusions were detected. In these studies, Wnt activation by RSPO3 fusion protein was clearly linked to increased tumorigenesis [26], although the role of native RSPO3, if any, remains unclear. Furthermore, the fact that RSPO3 fusions have not been reported in other cancers suggest that this particular phenomenon may be specific to colon cancer. With respect to other cancers, contribution of RSPO3 expression to patient survival has been addressed in one study, which reported a survival detriment in lung adenocarcinoma [31]. Our results appear to be at odds with these reports; however, we feel this contradiction highlights a potentially complex role for RSPO3 in cancer overall. In support of this notion, there is a report suggesting an opposite pattern of RSPO3 expression between adenocarcinoma and squamous cell carcinoma of the lung [32], even though the significance of this difference with respect to patient outcomes is unclear. It is also uncertain whether these observations are histopathology-specific or a phenomenon unique to the patient cohorts in question. One of the strengths of our study, thus, is a demonstration of consistent RSPO3 loss between normal prostate to prostate cancer, as well as a significant trend of decreased biochemical relapse-free survival with lower levels of RSPO3 expression in multiple patient cohorts. In combination with our functional in vitro and ex ovo investigations, our data strongly support a tumour-suppressor role for RSPO3 in human prostate cancer. The mechanistic basis for RSPO3 biology in prostate cancer, however, is still much less clear.

RSPO3 loss appears to increase a number of molecules (i.e. Twist/Snail) in the process of epithelial–mesenchymal transition (EMT). This process has been implicated in disease progression, development of metastatic castrate-resistant prostate cancer, and increased “stemness” in cancer progenitor niches [reviewed in 38, 39]. While investigating the precise mechanisms that may link RSPO3 with the process of EMT are beyond the scope of this paper, the increase in EMT markers is in concordance with the increase in invasiveness we observe in RSPO3 knockdown.

Given the literature on RSPO3 as a Wnt agonist [14, 15], it is possible that the increased invasiveness we observe in RSPO3-silenced tumours is due to decreased signaling through one of the Wnt pathways. However, investigation of Wnt activation by R-spondins is a challenging task given multiple RSPO family members and associated receptors, as well as several distinct but related Wnt pathways. For instance, despite identification of LGR proteins as receptors for the RSPO family in Wnt activation, studies now suggest that RSPO3 may act through LGR-independent mechanisms in activating Wnt [16, 40]. Furthermore, a large number of gene products are thought to be expressed as a result of both canonical and non-canonical Wnt signaling Wnt pathways [2], and any of these genes, alone or in combination, could constitute the mechanistic basis of RSPO3 action. Lastly, one cannot exclude a distinct signaling pathway not related to Wnt that is modulated by RSPO3. Thus, a more thorough investigation of the possible mechanism(s) of RSPO action is beyond the scope of this report. Recent reports reveal the complexity of the role Wnt signaling in prostate cancer, and suggest that this pathway may play a context-specific role in influencing cancer progression. With further studies exploring downstream signaling mechanisms of RSPO3, as well as contribution to other cancers, we may appreciate the full and seemingly complex role of RSPO3 in the future with an ultimate goal of capitalizing on its molecular profile as a biomarker or a therapeutic target.

## Additional file

**Additional file 1: Figure S1.** Schematic diagram of our search strategy for RSPO3 expression levels in patient cohorts. Oncomine Platform with the specified search keyword and applicable filters (left panel). PROGgene v2 with specified keyword and search parameters (right panel). **Figure S2.** Representative flow cytometry (histogram) plot for cell cycle analysis. DU145 cells transfected with control (DU145-siCtrl; left) or RSPO3-specific (DU145-siRSPO3; right) siRNA were collected and stained with propidium iodide as specified. Cell populations corresponding to different cell cycle phases were gated according to staining intensity, with percentages given above. Representative of three independent experiments.
