# Supplementary material for: RSPO3 is a prognostic biomarker and mediator of invasiveness in prostate cancer
Source: J Transl Med. 2019 Apr 15;17:125. doi: 10.1186/s12967-019-1878-3 (PMC6466739; doi:10.1186/s12967-019-1878-3)
Supplement: Supplementary file 1 — Additional file 1: Figure S1. Schematic diagram of our search strategy for RSPO3 expression levels in patient cohorts. Oncomine Platform with the specified search keyword and applicable filters (left panel). PROGgene v2 with specified keyword and search parameters (right panel). Figure S2. Representative flow cytometry (histogram) plot for cell cycle analysis. DU145 cells transfected with control (DU145-siCtrl; left) or RSPO3-specific (DU145-siRSPO3; right) siRNA were collected and stained with propidium iodide as specified. Cell populations corresponding to different cell cycle phases were gated according to staining intensity, with percentages given above. Representative of three independent experiments. [file 12967_2019_1878_MOESM1_ESM.pdf]

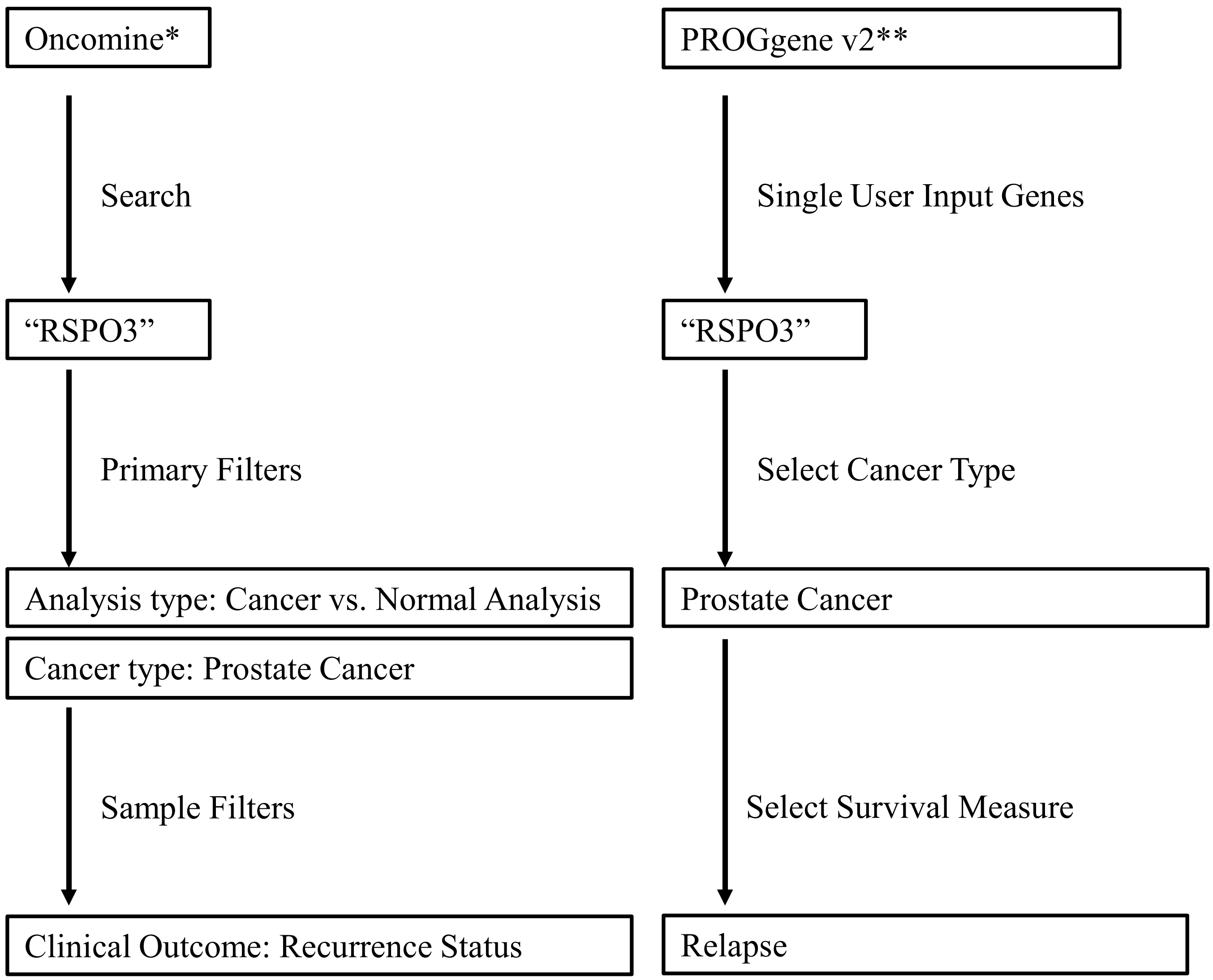

*www.oncomine.org

**http://watson.compbio.iupui.edu/chirayu/proggene/database/?url=proggene

Figure S1

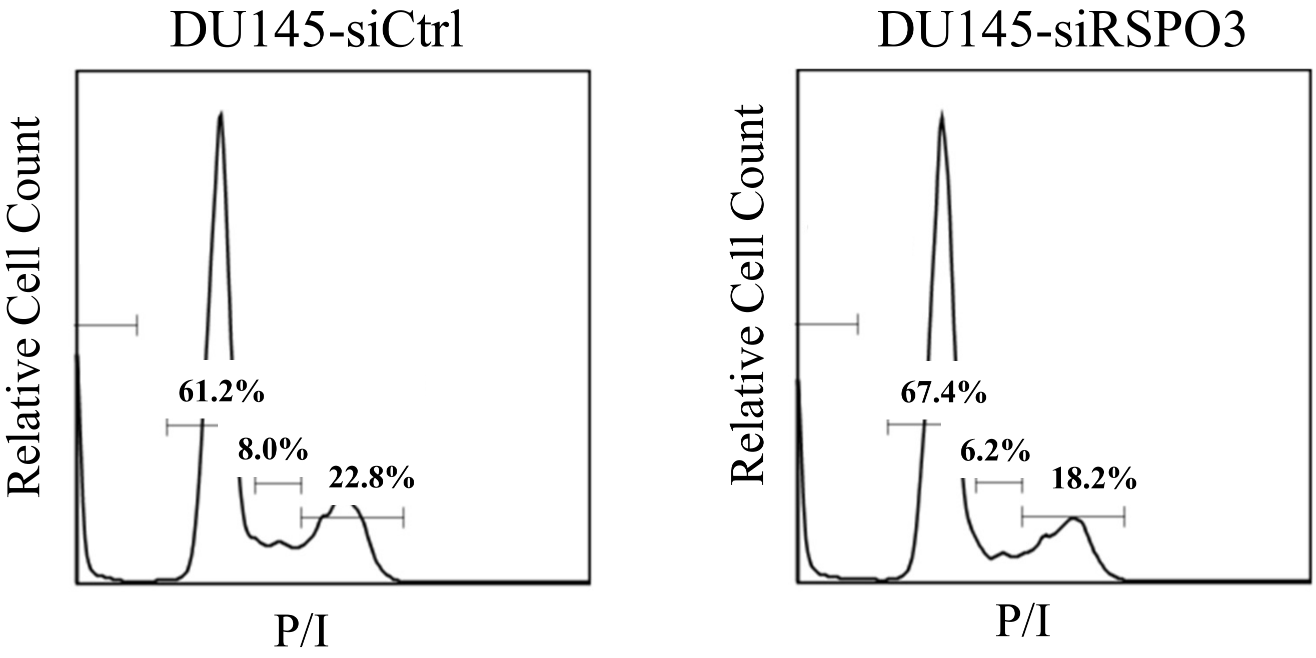
DU145-siCtrl
Relative Cell Count
61.2%
8.0%
22.8%
P/I
DU145-siRSPO3
Relative Cell Count
67.4%
6.2%
18.2%
P/I

Figure S2
